# Antibiotics Prescribing Pattern and Quality of Prescribing in Croatian Dental Practices—5-Year National Study

**DOI:** 10.3390/antibiotics13040345

**Published:** 2024-04-09

**Authors:** Lucija Petrac, Katarina Gvozdanovic, Vjera Perkovic, Nikolina Petek Zugaj, Neven Ljubicic

**Affiliations:** 1 School of Dental Medicine, University of Zagreb, 10000 Zagreb, Croatia; 2 Andrija Stampar Teaching Institute of Public Health, 10000 Zagreb, Croatia; 3 Department of Orthodontics, Faculty of Dental Medicine, University of Rijeka, 51000 Rijeka, Croatia; 4 Department of Internal Medicine, Clinical Hospital Sisters of Mercy, 10000 Zagreb, Croatia; neven.ljubicic@kbcsm.hr

**Keywords:** prescribing pattern, antibiotics, quality of prescribing, dentists, electronic prescription

## Abstract

Purpose: Antibiotic resistance is one of the biggest threats to global health today. The aim of this study was to analyze antibiotic prescribing patterns and quality of prescribing in Croatian dental practices over a 5-year period. Methods: This is a retrospective observational study based on the analysis of the electronic prescriptions (medicines in ATC groups J01 and P01) from dental practices in Croatia prescribed from 1 January 2015 to 31 December 2019. Prescriptions were retrieved from the Croatian Health Insurance Fund (HZZO). The analyses included the number of prescriptions, type and quantity of prescribed drugs, indication, and the patient’s and prescriber’s characteristics. Results: The consumption increased from 1.98 DID in 2015, to 2.10 DID in 2019. The most prescribed antibiotic was Amoxicillin with clavulanic acid followed by Amoxicillin, Clindamycin, Metronidazole and Cefalexin. The analyses showed that 29.79% of antibiotics were not prescribed in accordance with the contemporary guidelines for the proper use of antibiotics. Additionally, 22% of antibiotics were prescribed in inconclusive indications. Conclusion: The research showed an increase in antibiotic consumption over five years along with unnecessary prescribing of antibiotics in cases with no indications for its use. The development of national guidelines for antibiotic use is necessary.

## 1. Introduction

Over the past couple of decades, antimicrobial resistance has become one of the leading public health problems. It is estimated that 700,000 people die every year from antimicrobial-resistant bacterial infections and if antibiotic consumption does not decrease in the near future, it is predicted that 10 million people could die each year [1]. In 1998, the European Commission and the Dutch National Institute for Public Health and the Environment founded a European Antimicrobial Resistance Surveillance System (EARSS) [2]. Data from the European Surveillance of Antimicrobial Consumption Network (ESAC-Net) showed that there are great differences in antibiotic consumption around Europe, with lower consumption in northern parts of Europe and higher consumption in South Europe. During the years 2012 to 2016, a statistically significant decrease in antibiotic consumption was noted in Finland, Luxemburg, Norway, and Sweden while an increase in antibiotic consumption was seen in Greece and Spain [3]. Generally speaking, up to 11% of antibiotics were prescribed by dentists [4]. In dentistry, antibiotics are prescribed for prophylactic and therapeutic purposes [5]. In 2019, the American Dental Association (ADA) published new updated clinical recommendations and guidelines for antibiotic use which were based on general symptoms and signs of systemic infection as well as the availability of dentists and dental procedures in certain situations [6]. However, antibiotics prescribed for conditions such as caries, dry socket, pulpitis, localized marginal gingivitis and localized periapical infections, which could be treated with dental procedures, are indicative of the irresponsible use of antibiotics [7]. Considering the increased consumption of medications in general, there is a need for systemic monitoring of medication prescription and consumption. In the Republic of Croatia, monitoring of the total consumption of medication was implemented in 2014. Antimicrobial resistance has been observed in Croatia on a national level since 1996, and the monitoring of antibiotic consumption measured in Defined Daily Doses since 2000 [8]. Research conducted in Croatia in 2019, based on the data retrieved from an emergency public health dental clinic in Zagreb, revealed that antibiotics were prescribed in 48.8% of the cases treated, and their prescription was increased during the weekends and public holidays [9].

According to our knowledge, there is no research that comprises total antibiotic consumption on a national level in Croatia. Therefore, the aim of this study was to investigate antibiotic consumption in public health dental offices from 2015 until the end of 2019 in the Republic of Croatia as well as the correctness of the indication for which antibiotics had been used. 

## 2. Results 

During the period 1 January 2015 to 31 December 2019, a total of 1,583,088 e-prescriptions for antibiotics were prescribed by dentists in contractual primary oral health practices in the Republic of Croatia (public health dental offices). Prescriptions were issued for 1,215,022 individual patients and the number of prescribers slightly fluctuated from 2279 in 2015 to 2296 in 2019 (Table 1).

**Figure 1:**
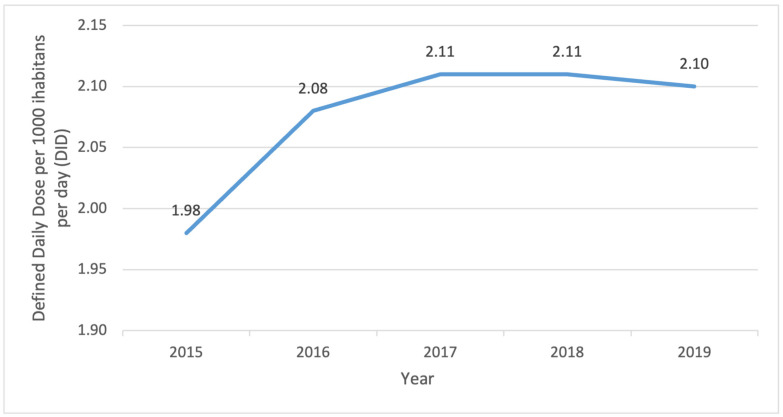
Antibiotic consumption in the Republic of Croatia over the period of five years.

From 2015 to 2019, the overall consumption of antibiotics expressed in DID was constant (Figure 1), with no notable trend over time (Spearman).

A total of 22 different antibiotics or antibiotic combinations were prescribed over time. The most prescribed antibiotic every year was amoxicillin with clavulanic acid which amounted to 75.2% of total antibiotic consumption at the end of the studied period (2019). In the same year, amoxicillin consumption accounted for 13.8% of total antibiotic consumption, clindamycin 4.8%, metronidazole 2.8% and cephalexin 1.4%. All other antibiotics were represented by less than 1% of the total consumption (Table 2).

Further analyses of the most used medicines revealed an increasing trend for amoxicillin/clavulanic acid and metronidazole and a decreasing trend for amoxicillin, clindamycin, and cephalexin.

Consumption of amoxicillin/clavulanic acid was 1.41 DID in 2015 and has been steadily increasing year by year (up to 1.58 in 2019). Except for the amoxicillin/ clavulanic acid combination, a statistically relevant increasing trend in consumption was noted for metronidazole. For clindamycin and cephalexin, there is a decreasing trend in use while for amoxicillin alone, no trend over time has been noted. 

### 2.1. Patients

The largest antibiotic consumption was for the patients in the age group 18–65 years; however, no trend over time was noted (the same as for the age group 0–6 years). For the age group 7–17 years, there was a decreasing trend in antibiotic use and the only increasing trend was noted in the age group 65+ (395,294.3 DDDs in 2015 to 489,809.5 in 2019, *p* = 0.0018) (Table 3). 

877,673 prescriptions were prescribed to women and 705,415 to men. The difference was statistically significant (*p* < 0.05). The average patient age was 49.6 years (median age was 51 years) (Table 4).

### 2.2. Indications

Every prescription contained information about the indication for which the medicine was prescribed. All the indications were divided into three groups: correct indication for antibiotic use, incorrect indication for antibiotic use and indications where antibiotics may or may not be prescribed (depending on a clinical situation). The most common indication was Periapical abscess without sinus (K04.7) with close to 20% of all prescriptions during the studied period (Table 5). The second most common indication was Diseases of pulp and periapical tissues (K04) and the third was Chronic apical periodontitis (K04.5). Of all the prescribed antibiotics, 48.31% were prescribed for correct indications. However, 0.05% of them were prescribed for correct indications but were administered for indications that were not related to dentistry. 

### 2.3. Settlement Type

The consumption rates in urban areas were higher than in rural areas (*p* < 0.05). Antibiotic consumption in urban areas was increasing year by year, from 1.71 DID in 2015 to 1.82 DID in 2019, and in rural areas from 0.26 DID in 2015 to 0.27 DID in 2019. Indicating a 6% increase in urban areas and a 5% increase in rural areas during the observed period (Table 6).

### 2.4. Weekdays

The highest prescription rates for antibiotics were on Mondays and Fridays. Up to 25% of all antibiotics were prescribed on Mondays, and up to 20% of all antibiotics were prescribed on Fridays (Table 7).

Out of all prescribed antibiotics, 29.79% were prescribed for incorrect indications. Among them, 0.11% of antibiotics were given for diagnoses unrelated to dentistry (Table 8). 

Table 9 shows a list of indications for which antibiotics may or may not be prescribed, and that percentage amounts to nearly 22% of the total prescribed antibiotics.

## 3. Discussion

### 3.1. Antibiotic Consumption 

This national study showed an increase in antibiotic consumption in dentistry in an observed period. This is in accordance with a previous study from Croatia [10]. In Croatia, the increase in antibiotic consumption in dentistry follows the trend of increased consumption of medication in general. During the period 2015 to 2019, there has been a trend of increased medication consumption measured in DID with an average increment of 6.6% a year [11]. Klein et al. investigated global antibiotic consumption and came to the conclusion that there had been an increase of 65% between the years 2000 and 2015 [12]. DDD grew from 21.1 billion to 34.8 billion while the rate of antibiotic consumption increased 39% from 11.3 to 15.7 DDD per 1000 inhabitants daily during the observation period. An increase in antibiotic consumption was noted even in the Czech Republic between the years 2006 and 2012 [13]. Medication prescribed by dentists was increased even in Norway by 30% and Australia by 264% from 2005 to 2016 [14,15]. According to data from the European Antimicrobial Resistance Surveillance Network in systemic consumption from 2010 to 2019, total consumption of antibiotics was decreased, even though six countries (Bulgaria, Greece, Iceland, Ireland, Latvia, and Poland) showed a statistically significant increase in antibiotic consumption [16]. Interestingly, in Croatia from 2015 to 2019, the number of prescribers increased while the population number decreased. On the other hand, different research that compared antibiotic prescription by dentists between England, Scotland, Norway, and Sweden showed a decrease in antibiotic prescribing trend from 2010 to 2016: in Sweden, it was lower by 19%, England by 18%, Scotland by 13% and Norway by 4% [17]. Additionally, research conducted in Germany and Belgium showed the same trend [18,19]. Palmer et al. pointed out a possible explanation for an overprescription of antibiotics in dentistry—inaccurate dental diagnoses (indications) with an aim to prevent the further spreading of infection, the pressure of time or postponed treatment [20]. This could be related to a lack of patient cooperation, dentists’ work overload and prescribing an antibiotic “just in case” before the weekend or a vacation [21,22]. Yingling et al. investigated these reasons among the members of the American Association of Endodontists [23]. Part of the members claimed that they prescribed antibiotics for “medical–legal” reasons, so they would not lose a patient or their recommendation. One of the reasons for antibiotic prescription was pressure from the patient or general dentist who referred them to an endodontist specialist and required antibiotics to be prescribed for every endodontic treatment. Al-Khatib investigated, among Jordanian dentists, how their patients take antibiotics before dental procedures without even consulting them. Results of this study showed that 33.9% of dentists claimed that more than once a week they were pressured by patients to prescribe antibiotics even though there were no indications for it, and 22% of dentists claimed they were under pressure by parents who demanded an antibiotic for their children even though there were no indications for prescribing [24].

### 3.2. Indications

In this study, most antibiotics were prescribed for the following diagnoses according to the ICD classification: Periapical abscess without sinus (K04.7), Diseases of pulp and periapical tissues (K04) and Chronic apical periodontitis (K04.5). Periapical abscess without sinus was one of the most often used diagnoses for antibiotic use in Turkey [25]. Comparable results were seen in Belgium and Kosovo [26,27]. This study also revealed that diagnoses such as Acute periodontitis (K05.2) and Chronic periodontitis (K05.3) were used in less than 5% of cases when antibiotics were prescribed. However, studies showed that in Italy and France, pericoronitis was a diagnosis used in more than 50% of the cases in antibiotic prescriptions [28,29]. According to the guidelines of the European Society of Endodontology, indications for systemic antibiotic treatment in conjunction with endodontic therapy are indicated in: acute apical abscess in medically compromised patients, acute apical abscess with systemic involvement (localized fluctuant swellings, elevated body temperature > 38 °C, malaise, lymphadenopathy); trismus; progressive infections (rapid onset of severe infection in less than 24 h, cellulitis or a spreading infection, osteomyelitis) where onward referral to oral surgeons may be necessary; replantation of avulsed permanent teeth. In these cases, topical administration of antibiotics may also be indicated. Soft tissue trauma requiring treatment (e.g., sutures, debridement).

Clinical diagnoses where antibiotics are not indicated are: Symptomatic irreversible pulpitis (pain, with no other symptoms and signs of infection); Pulp necrosis; Symptomatic apical periodontitis (pain, pain to percussion and biting and widening of periodontal ligament space); Chronic apical abscess (teeth with sinus tract and periapical radiolucency); Acute apical abscess without systemic involvement (localized fluctuant swellings) [30]. Considering the indication for correct prescription of antibiotics, their use for diagnoses such as caries (ICD K02), pulpitis (K04), retained dental root (K08.3), pulp necrosis (K04.1), dentofacial anomalies (K07), encounter for dental examination (Z01.2), deposits (accretions) on teeth (K03.6), presence of dental prosthetic device (Z97.2) points towards ignorance of dentists in Croatia on the correct use or prescription of antibiotics. This correlates with another national research, where results showed that antibiotics were prescribed in clinical situations where they were not supposed to like pulpitis in 25.6% of cases and cavity in 9.3% of cases [9]. Similar results were seen in research in other countries such as D’Ambrosio et al., where antibiotics were used for pulpitis (19.4% of cases) [28]. Meanwhile, in India, Malaysia, Saudi Arabia and Cambodia, antibiotics were given in 26.7% of the cases for pulpitis [31]. Different research in the United Kingdom (UK) interestingly pointed out that in 75% of dental cases, antibiotics were prescribed incorrectly [32]. Another piece of research conducted in Croatia investigated the attitudes of dentists towards antibiotic prescription and the results pointed out that their habits of prescribing are not in line with contemporary guidelines [33]. Additionally, they showed ignorance when prescribing antibiotics for immunocompromised patients. It is alarming that there was an increase in antibiotic prescriptions for inaccurate diagnoses in Croatia in the observed period. Prescribed antibiotics in the observed period for diagnosis of pulpitis were increased by 4.1%, dental caries by 39.1%, caries of dentine by 13.5%, caries of cementum by 3.2%, other dental caries by 3.0%, retained dental root by 2.9%, dental examination 144.7%. This study revealed antibiotics were prescribed in 29.79% of cases for incorrect indications and in nearly 22% of cases for diagnoses where we cannot determine with certainty whether antibiotics were necessary or not. These indications depend greatly on the clinical situation, or a very broad indication was used instead of a specific clinical diagnosis. 

In the period 2015 to 2019, diagnosis of acute apical periodontitis of pulpal origin was in the top five most used diagnoses for the prescription of antibiotics. According to the American Dental Association, antibiotics can be prescribed for this diagnosis only in cases when conservative treatment is not available within 24 h, or if symptoms progression is noted [6]. Because we do not have data that could confirm if dental care was available within 24 h or not, we cannot verify if antibiotic use in these cases was appropriate. National guidelines and their implementation have an impact on the rational use of antibiotics, research from Sweden has shown [34]. Since Croatia does not have national guidelines for prescribing antibiotics, that could potentially explain the reasoning for the incorrect use of antibiotics. Certain indications and guidelines for drug prescription published online from the Croatian Health Insurance Fund webpage do not fully correspond with the guidelines presented in the summary of products. Also, there are limitations within the computer software, where appropriate diagnosis could not be found, which could also lead to incorrect use of diagnosis for antibiotic prescription, even though clinical situation requires it. Furthermore, we do not have the data about if and when antibiotics were used for prophylactic purposes. 

One of the problems when comparing data from different research is the fact that not all dentists use the same diagnosis classification. More unified classification would make comparison easier. 

In this research, the most prescribed antibiotic in the observed period was amoxicillin with clavulanic acid (71%). This correlates with another national piece of research in Croatia [9,10,35]. Following it are amoxicillin, clindamycin, metronidazole, cephalexin, and azithromycin. Their consumption accounted for 96% of the total consumption of all prescribed antibiotics by public health service dentists. The consumption of amoxicillin with clavulanic acid increased by 11.4% during the observed period, metronidazole by 15.5%, and azithromycin by 4.8%, while the consumption of amoxicillin decreased by 6%, clindamycin by 9.9%, cephalexin by 42.7%, and clindamycin by 9.9%. The study in the Czech Republic also showed an increased consumption of clindamycin and amoxicillin with clavulanic acid by 60% from 2006 to 2012 [13]. Unlike the research in Croatia, where the use of amoxicillin with clavulanic acid is the first choice of antibiotics prescribed, in Belgium, during the period from 2000 to 2016, the most prescribed antibiotic was amoxicillin, followed by amoxicillin with clavulanic acid, clindamycin, clarithromycin, doxycycline, azithromycin, and metronidazole [19]. Similar results were shown in a study conducted in Australia, where the most frequently prescribed antibiotic was amoxicillin, followed by amoxicillin with clavulanic acid and metronidazole [15]. These three antibiotics accounted for more than 80% of all antibiotics prescribed by dentists. A study conducted in Germany also showed that amoxicillin is the first choice of antibiotic for treating dentoalveolar infections [36]. A study conducted in the United States of America has shown that the most prescribed antibiotics were amoxicillin followed by penicillin V and clindamycin for individuals allergic to penicillin [37]. Research indicated that amoxicillin remains the most used antibiotic in the treatment of odontogenic infections worldwide [7,20,38,39]. Dominguez et al. stated in their research that dentists with more experience prescribed amoxicillin with clavulanic acid as their first choice in treatment, while those with less experience prescribed only amoxicillin [40]. According to the guidelines of the European Society of Endodontology, amoxicillin is the first-choice antibiotic for treating infections in non-allergic and non-immunocompromised patients [30]. Choosing amoxicillin with clavulanic acid as the first choice in treating infections by dentists with more work experience could be associated with a lack of education and adherence to new guidelines. The research conducted in Croatia showed similar results. Dentists with less experience showed better knowledge of the proper use of antibiotics [41]. A study by Smith et al. indicated that phenoxymethylpenicillin is more commonly prescribed in Norway and Sweden, while broad-spectrum amoxicillin is predominantly prescribed in England and Scotland [17]. The previous guidelines in England had recommended amoxicillin as the first-choice antibiotic, unlike the recommendations of the Norwegian Institute of Public Health, which recommends the use of phenoxymethylpenicillin as the first-choice antibiotic in acute dentoalveolar processes. In the new guidelines in England, phenoxymethylpenicillin is now listed as the first-choice antibiotic [42,43,44]. In Croatia, the consumption of phenoxymethylpenicillin prescribed by dentists amounted to less than 1%. Metronidazole is useful for treating anaerobes, and recommendations suggest its use in combination with penicillin for the treatment of odontogenic infections [45]. Considering the use of metronidazole in combination with penicillin, the increase in metronidazole consumption parallels the increase in the consumption of amoxicillin with clavulanic acid. The highest prescription rates for antibiotics occur on Mondays and Fridays. The fact that the highest antibiotic consumption is on Mondays and Fridays could be linked to the availability of dental services. In addition to the mentioned availability of dental services, the reason could also be the pressure from patients to prescribe antibiotics “just in case”, especially if they are planning to travel, and in similar situations. Public dental offices in Croatia operate under specific contractual terms, including a designated number of working Saturdays, and they are closed on Sundays. This aligns with the observed trend of increased antibiotic prescriptions on Fridays and Mondays. The study conducted by Kuehlein et al. in Germany in 2010 also showed an increased prescription of antibiotics on Fridays and Mondays [46]. 

This study shows increased consumption rates in both urban and rural areas of the Republic of Croatia in the period 2015 until the end of 2019. In urban areas, the consumption rate was 1.82 DID while in rural areas, it was 0.27 DID, indicating a 6% increase in urban areas and a 5% increase in rural areas during the observed period. The consumption of antibiotics was statistically higher in urban areas than in rural areas (*p* < 0.05). The study conducted in Croatia in 2018 on parents’ knowledge regarding the use and resistance of antibiotics in rural and urban areas showed that, despite a higher level of knowledge about antibiotics among parents in urban areas, it did not influence antibiotic consumption levels [47]. In contrast, a study in the UK has shown higher antibiotic prescriptions in less developed areas, indicating a higher incidence of caries and other dental diseases in those regions [48]. Considering that untreated caries can lead to abscesses, prompting patients to visit a dentist, this could be a contributing factor to the higher antibiotic prescriptions in those areas [49]. Manski et al., in their studies, emphasize that factors such as income, education, and health insurance could be correlated with individuals using dental care. However, they acknowledge that these factors alone cannot explain the usage patterns for all individuals [50]. A study conducted in the United States of America (USA) has shown that adults in rural areas have a 65% lower chance of receiving adequate preventive dental care compared to those in urban areas. Contributing factors to this result may include a shortage of dentists in those areas, educational disparities, and the fact that health is not a priority due to other essential needs, such as food, particularly for individuals with lower socioeconomic status [51]. One of the reasons that could explain the higher usage of antibiotics in urban areas could be the fact that more people live in urban areas rather than in rural areas. 

### 3.3. Patients

In this research, from 2015 until the end of 2019, more antibiotics were prescribed to female patients. Similar results were noted in previous research by Peric et al. from 2015, wherein in 54 % of cases, antibiotics were prescribed to women and in 46% of cases to men [35]. Such results are in accordance with research conducted in Norway (2010–2016) and the United States of America [52,53]. Similar results were also seen in a study in Sweden where women were prescribed more antibiotics than men in the age group 21–80 years [34]. On the contrary, Lipsky et al., summarizing contemporary literature on differences in oral health in men and women, stated that men have poorer oral hygiene habits, a higher prevalence of periodontal diseases, and that they more often than women seek dental help for acute problems rather than for preventive reasons [54]. Speaking of preventive dentistry, Lund et al. concluded that there is less chance for antibiotic prescription if a patient has a dental appointment once a year or more often [34]. Considering that a study has shown that women have a more positive attitude towards oral health, better behavior related to oral health and visit dentists more frequently, it is not known why more antibiotics are prescribed to women [54]. 

In this research, the highest consumption was recorded for the age group 18–65 years. Similar results could be found in another study in Turkey where most of the antibiotics prescribed were for the age group 18–64 years [55]. However, in the age group 7–17 years, there was a statistically significant decrease in antibiotic consumption, while in the group 65+ years, there was a statistically significant increase. In 2017, a national program was implemented in the Republic of Croatia [56]. The program consists of obligatory preventative dental exams before children are enrolled in kindergarten, first grade in elementary school (age 7) and sixth grade of elementary school (age 12). This could explain the decrease in antibiotic consumption in the age group 7–17 years because a written confirmation from a dentist is needed to enroll in school which obligates parents to bring their children to regular dental appointments thus giving them the opportunity to avoid clinical situations that would require antibiotic use. An increase in antibiotics in the age group 65+ years could be linked to other comorbidities and dentists’ decision to use antibiotics to prevent complications considering patients’ age, and whether there is an indication for antibiotic use or not. 

Since the overuse of antibiotics is in direct connection with antimicrobial resistance, it is important to mention that in some works of research, the highest frequency of resistance was for amoxicillin, clindamycin, and metronidazole [57]. Even though the resistance patterns were widely variable and have not yet reached critical levels, it is paramount for dentists to follow available guidelines on antibiotic use in certain clinical situations, as well as for patients to be educated on antibiotic use and resistance. 

## 4. Materials and Methods 

This is a retrospective observational study based on the analysis of the electronic prescriptions (ePrescription) for antibiotics from all primary dental practices in Croatia (public health care dental offices). E-prescriptions for the period 1 January 2015 to 31 December 2019 were retrieved from the Croatian Health Insurance Fund (CHIF) and they included only prescriptions for the medicines from ATC groups J01 and P01 (as per Anatomical–Therapeutic–Chemical Classification (ATC) developed by the World Health Organization) [58]. The Ethics Committee of the School of Dental Medicine, University of Zagreb (protocol code 05-PA-30-XIV-2/2020, 17 February 2020) approved this study, meaning approved the retrieval of data from the public health insurance server. 

The analysis included the number of prescriptions per year, type and quantity of prescribed drugs, trends in antibiotic use, indication for prescribing antibiotics, number of prescribers and patient’s characteristics (gender, age) and settlement type. Our research comprised numerical and categorical variables. Numerical variables, also known as quantitative variables, represent measurements and can take on values within a given range, such as DDDs, population, etc. On the other hand, categorical variables, also known as qualitative variables, describe attributes or qualities and therefore represent distinct categories or groups to which data can be assigned, in our case sex, settlement type, etc.

Individual patients and prescribers are uniquely coded in every prescription—the same prescriber will have the same code for every prescription issued over a studied period. Every e-prescription contained an indication for prescribing (a diagnosis), coded according to the International Classification of Diseases and Related Health Problems (ICD-10) [59]. A total of 1,583,088 e-prescriptions for antibiotics were prescribed and prescriptions were issued for 1,215,022 individual patients. Inclusion criteria were all antibiotic prescriptions made by dentists in a public health dental office. There were no exclusion criteria. 

Antibiotic consumption is expressed in Defined Daily Dose (DDDs) per 1000 inhabitants per day (DID) [60]. Data about the number of inhabitants and categorization of rural and urban areas, for this period, were taken from published data by the World Bank [61].

For the analysis purpose, patients were divided into four age groups: 1–6 years (pre-school), 6–17 years (school children), 18–65 years (working population), and 65+ years (retired population). 

This study employed linear regression and Spearman’s rank correlation coefficient for trend analysis. The chi-square test was utilized to compare medication prescriptions in urban versus rural areas. Additionally, differences in average consumption across years between urban and rural areas, as well as between genders, were analyzed using the Mann–Whitney U test. Statistical significance was determined at a *p*-value threshold of <0.05.

## 5. Limitations of the Study 

This study investigated all prescriptions from public health dental practices; however, data from private dental practices and prescriptions from private dental practices are not included and therefore were not considered in this study. According to the data, prescriptions from private practices amounted to 11% of the total consumption of medicines in the Republic of Croatia, in contrast to the total consumption of medicines prescribed in public health dental practices, which amounted to 89% [62]. Additionally, unmeasured factors, such as patient’s disease severity, coexisting comorbidities, and combined medication use, may also influence the results in total antibiotic consumption and antibiotic prescribing patterns of dental practitioners. Data may be generalized only to the public health general dentists in Croatia because of the difference in the use of prescribed antibiotics between public and private dentists. In addition, the results are less generalizable to other hospitals and countries.

## 6. Conclusions

Based on the data from this study, from 2015–2020, regarding antibiotic consumption, we can conclude the following:

- consumption of antibiotics prescribed by public health general dentists has increased over a 5-year period
- the most prescribed antibiotics were amoxicillin with clavulanic acid, amoxicillin, clindamycin, metronidazole and cephalexin
- 29.79% of antibiotics were prescribed for clinical diagnoses that do not have an indication for antibiotic use
- more antibiotics were prescribed to women
- the largest antibiotic consumption was in the age group 18–65 years
- consumption rates in urban areas were higher than in rural areas

Finally, since the possible reasons for antibiotics being inappropriately prescribed were pressure from the patient, lack of dentists’ compliance with established guidelines, and lack of dentists’ knowledge like in the case of immunocompromised patients we can conclude that additional education for dentists regarding antibiotic use is necessary as well as raising awareness among both dentists and patients on the growing global problem of antimicrobial resistance.

The development of national guidelines for antibiotic use is essential for rational antibiotic use. 

## Figures and Tables

**Table 1:** Overview of number of prescribers, prescriptions, and consumption of antibiotics from 2015 to 2019.

Year	Number of Individual Prescribers	Number of Prescriptions	Number of Individual Patients	Defined Daily Dose per 1000 Inhabitants per Day (DID)
2015	2279	314,425	242,563	1.98
2016	2236	322,983	247,861	2.08
2017	2249	319,695	245,210	2.11
2018	2261	316,108	241,840	2.11
2019	2296	309,877	237,548	2.10

**Table 2:** Consumption/percentage of total antibiotic consumption per year for five most used antibiotics/antibiotic combinations from 2015 to 2019.

	2015		2016		2017		2018		2019		
INN (International Non-Proprietary Name)	DDDs	% of Total Consumption	DDDs	% of Total Consumption	DDDs	% of Total Consumption	DDDs	% of Total Consumption	DDDs	% of Total Consumption	*p* Value (Lin Reg)
amoxicillin/clavulanic acid	1.41	71.46	1.50	72.20	1.54	73.30	1.57	74.32	1.58	75.16	0.019
amoxicillin	0.31	15.60	0.32	15.36	0.31	14.78	0.30	14.24	0.29	13.86	0.103
clindamycin	0.11	5.66	0.11	5.48	0.11	5.14	0.10	4.94	0.10	4.80	0.018
metronidazole	0.05	2.66	0.06	2.68	0.06	2.72	0.06	2.80	0.06	2.90	0.001
cephalexin	0.05	2.54	0.05	2.3	0.04	2.02	0.03	1.62	0.03	1.38	0.002

**Table 3:** Antibiotics consumption by the age group in DDDs from 2015 to 2019.

Age Group	2015	2016	2017	2018	2019
0–6	48,841	49,520.87	47,189.11	47,067.38	47,342.23
7–17	205,161.5	199,768.1	189,813.9	179,900.7	171,925.1
18–65	2,384,872	2,478,907	2,464,372	2,451,860	2,403,535
65+	395,294.3	430,236.4	455,300.1	471,721.2	489,809.5

**Table 4:** Number of antibiotics prescriptions by gender and percentage of antibiotic prescriptions to women and men from 2015 to 2019.

Year	Number of Antibiotics Prescriptions by Gender		Percentage of Antibiotics Prescription	
	Women	Men	Women	Men
**2015**	173.691	140.734	55.24%	44.76%
**2016**	178.527	144.456	55.27%	44.73%
**2017**	177.565	142.13	55.54%	44.46%
**2018**	175.393	140.715	55.49%	44.51%
**2019**	172.497	137.38	55.67%	44.33%

**Table 5:** List of the most common indications used in the prescriptions.

		2015	2016	2017	2018	2019
Indication (ICD 10 Code)	Indication	Percentage Out of Total Drug Prescription				
K04.7	Periapical abscess without sinus	20.16	20.55	20.12	19.55	19.18
K04	Diseases of pulp and periapical tissues	15.64	14.65	14.62	14.87	14.62
K04.5	Chronic apical periodontitis	12.44	12.74	12.82	12.58	12.27
K04.1	Necrosis of pulp	8.15	7.94	7.52	7.06	6.79
K04.4	Acute apical periodontitis of pulpal origin	6.56	6.69	6.96	6.82	6.48
K04.0	Pulpitis	5.12	5.30	5.28	5.40	5.40
K05.2	Acute periodontitis	4.14	4.20	4.23	4.21	4.27
K10.2	Inflammatory conditions of jaws	3.46	3.36	3.38	3.46	3.49
K08.3	Retained dental root	2.69	2.82	2.92	2.79	2.81
K05.0	Acute gingivitis	2.06	2.19	2.32	2.38	2.39
K02	Dental caries	1.86	2.03	2.26	2.39	2.62
K05	Gingivitis and periodontal diseases	1.64	1.59	1.67	1.65	1.64
K05.3	Chronic periodontitis	1.60	1.54	1.50	1.48	1.53
K10.3	Alveolitis of jaws	1.39	1.42	1.35	1.42	1.46
K05.4	Periodontosis	1.32	1.39	1.42	1.40	1.40

**Table 6:** Antibiotic consumption in urban and rural areas from 2015 to 2019 expressed in DID (Defined Daily Dose per 1000 inhabitants per day).

	2015	2016	2017	2018	2019
Settlement Type	DID	DID	DID	DID	DID
**Urban**	1.718105	1.8066	1.82592	1.838194	1.821819
**Rural**	0.265538	0.276404	0.28063	0.279353	0.279551

**Table 7:** Percentage of antibiotics prescribed by days of the week in the period 2015 to 2019.

	2015	2016	2017	2018	2019
**Monday**	24.33	23.78	23.76	23.38	24.42
**Friday**	18.89	19.23	19.41	19.57	19.43
**Tuesday**	18.51	18.57	18.13	18.31	17.88
**Wednesday**	17.91	17.45	17.87	17.67	17.60
**Thursday**	17.28	17.91	17.77	17.93	17.54
**Saturday**	2.61	2.62	2.59	2.66	2.80
**Sunday**	0.46	0.45	0.48	0.47	0.34

**Table 8:** List of indications in which antibiotic prescriptions are not indicated.

		Percentage Out of Total Drug Prescription					
Indication (ICD 10 Code)	Indication	2015	2016	2017	2018	2019	Total
**K04.1**	**Necrosis of pulp**	8.1513875	7.94376175	7.51904159	7.064674099	6.79108162	7.50343246
**K04.0**	**Pulpitis**	5.1169595	5.30492317	5.2847245	5.396573323	5.4047251	5.30514123
**K08.3**	**Retained dental root**	2.6906257	2.81531845	2.91527862	2.792716413	2.81014725	2.807220934
**K05.0**	**Acute gingivitis**	2.0593146	2.18927931	2.32033657	2.375137611	2.39385304	2.268710381
**K02**	**Dental caries**	1.8589489	2.0344724	2.26278171	2.388740557	2.62297621	2.233247954
**K02.1**	**Caries of dentine**	0.9162757	0.93627219	0.99375968	1.028445974	1.05558012	0.986373831
**Z01.2**	**Dental examination**	0.8574382	0.83162272	0.61152036	1.443493996	2.1285865	1.169185489
**K01.1**	**Impacted teeth**	0.8418542	0.82109585	0.81390075	0.798777633	0.79902671	0.81557262
**K01**	**Embedded and impacted teeth**	0.8151387	0.90035698	0.99125729	1.028762322	1.08365577	0.96399647
**K08.1**	**Loss of teeth due to accident, extraction or local periodontal disease**	0.8103681	0.9006666	0.98093495	1.013893986	1.13722542	0.968547798
**K08**	**Other disorders of teeth and supporting structures**	0.7146378	0.66969469	0.61777632	0.618143166	0.63638153	0.65178804
**K00.7**	**Teething syndrome**	0.5960086	0.58331243	0.5874349	0.545383223	0.49309887	0.561836107
**K01.0**	**Embedded teeth**	0.3994593	0.3854692	0.44667574	0.403343161	0.42855714	0.412906554
**K10**	**Other diseases of jaws**	0.3473006	0.29258506	0.25712007	0.261619447	0.27010717	0.285911871
**K00.6**	**Disturbances in tooth eruption**	0.2808301	0.30589845	0.29684543	0.267313703	0.27075259	0.284710826
**K04.8**	**Radicular cyst**	0.26461	0.26967364	0.26181204	0.237893378	0.26107133	0.259236034
**K03.6**	**Deposits [accretions] on teeth**	0.2229466	0.26286213	0.23929057	0.252761714	0.30044179	0.255696113
**S02.5**	**Fracture of tooth**	0.2213564	0.20341628	0.24648493	0.24675111	0.26494383	0.236542609
**K00**	**Disorders of tooth development and eruption**	0.2134054	0.18329138	0.22490186	0.203095145	0.18684833	0.202470865
**K08.0**	**Exfoliation of teeth due to systemic causes**	0.1895524	0.19443748	0.21989709	0.230933732	0.29463303	0.225669993
**K04.2**	**Pulp degeneration**	0.0995468	0.10433986	0.11354572	0.114834171	0.08519509	0.103669129
**K05.5**	**Other periodontal diseases**	0.0865071	0.08483419	0.10291059	0.085413846	0.09874886	0.091721894
**K08.8**	**Other specified disorders of teeth and supporting structures**	0.0845989	0.09907642	0.10228499	0.07307629	0.07260945	0.086538437
**K03**	**Other diseases of hard tissues of teeth**	0.0833267	0.06068431	0.06130843	0.063269515	0.05970111	0.065678186
**K09**	**Cysts of oral region, not elsewhere classified**	0.077602	0.06161315	0.06912839	0.058207954	0.06228278	0.065804612
**Z01**	**Other special examinations and investigations of persons without complaint or reported diagnosis**	0.0709231	0.08885917	0.03972536	0.027522239	0.03259358	0.052150629
**K02.0**	**Caries limited to enamel**	0.069651	0.06997272	0.06193403	0.064218558	0.07551383	0.068269914
**K02.2**	**Caries of cementum**	0.0594736	0.06842465	0.05849325	0.04966657	0.06228278	0.059736175
**K08.9**	**Disorder of teeth and supporting structures, unspecified**	0.0550211	0.04829976	0.04066376	0.040176142	0.04776089	0.046398256
**K02.8**	**Other dental caries**	0.0429355	0.04365555	0.04691972	0.043972313	0.04485651	0.04450187
**K06**	**Other disorders of gingiva and edentulous alveolar ridge**	0.0400731	0.04117864	0.04066376	0.043339618	0.04743818	0.04254227
**K07.3**	**Anomalies of tooth position**	0.039119	0.04272671	0.034095	0.046819441	0.04292026	0.041151587
**K05.6**	**Periodontal disease, unspecified**	0.039119	0.03405752	0.0328438	0.028471282	0.03065733	0.033060338
**K06.2**	**Gingival and edentulous alveolar ridge lesions associated with trauma**	0.0302139	0.03189022	0.03034142	0.037645362	0.03452983	0.032933912
**K02.3**	**Arrested dental caries**	0.0263974	0.02043451	0.02627504	0.016450074	0.01613543	0.021176316
**K12.0**	**Recurrent oral aphthae**	0.023217	0.01919606	0.02252147	0.015501031	0.01710356	0.019532781
**K09.2**	**Other cysts of jaw**	0.0225809	0.01207494	0.01876789	0.021511635	0.02065336	0.019090291
**K07**	**Dentofacial anomalies [including malocclusion]**	0.0225809	0.02569795	0.02596225	0.02878763	0.02872107	0.026359772
**K04.9**	**Other and unspecified diseases of pulp and periapical tissues**	0.0206727	0.01857683	0.02158307	0.026889544	0.03065733	0.023641618
**K00.9**	**Disorder of tooth development, unspecified**	0.0203546	0.01455185	0.02033188	0.020562593	0.01807169	0.018774226
**K03.9**	**Disease of hard tissues of teeth, unspecified**	0.0200366	0.01981528	0.02095748	0.014551989	0.01613543	0.018331736
**K07.6**	**Temporomandibular joint disorders**	0.0165381	0.02724602	0.01782949	0.023093373	0.02033065	0.02104989
**K09.0**	**Developmental odontogenic cysts**	0.015266	0.0157903	0.00938394	0.012021208	0.01064939	0.012642577
**K06.0**	**Gingival recession**	0.0149479	0.0089788	0.01188633	0.013919293	0.01774898	0.013464344
**K00.8**	**Other disorders of tooth development**	0.0146299	0.01145571	0.00563037	0.006959647	0.00774501	0.009292294
**K02.9**	**Dental caries, unspecified**	0.0146299	0.01238455	0.01188633	0.012653903	0.01452189	0.013211493
**K04.3**	**Abnormal hard tissue formation in pulp**	0.0146299	0.01176533	0.0165783	0.010123122	0.01226293	0.013085067
**K00.0**	**Anodontia**	0.0139938	0.01393262	0.01063514	0.012337556	0.00935855	0.012073661
**K06.1**	**Gingival enlargement**	0.0139938	0.01331339	0.01845509	0.013602946	0.01387647	0.014665389
**K09.9**	**Cyst of oral region, unspecified**	0.0136758	0.01269417	0.01438871	0.013286598	0.01516731	0.013843621
**K03.0**	**Excessive attrition of teeth**	0.0108134	0.01238455	0.01501431	0.014551989	0.01161751	0.012895428
**K06.8**	**Other specified disorders of gingiva and edentulous alveolar ridge**	0.0104953	0.01176533	0.00656876	0.010123122	0.00774501	0.009355507
**K03.1**	**Abrasion of teeth**	0.0092232	0.01083648	0.00969674	0.009806775	0.00839043	0.009608358
**K00.1**	**Supernumerary teeth**	0.0089051	0.00866919	0.00875835	0.004112519	0.0038725	0.006890204
**K10.9**	**Disease of jaws, unspecified**	0.007633	0.01021726	0.00938394	0.01170486	0.01355376	0.010493339
**K10.1**	**Giant cell granuloma, central**	0.0069969	0.00433459	0.01063514	0.005694256	0.0074223	0.00701663
**K07.4**	**Malocclusion, unspecified**	0.0063608	0.00743073	0.00719436	0.005061561	0.00903584	0.00701663
**K08.2**	**Atrophy of edentulous alveolar ridge**	0.0060428	0.00433459	0.00719436	0.006643299	0.00677688	0.006194863
**K10.0**	**Developmental disorders of jaws**	0.0060428	0.00247691	0.00250239	0.004428866	0.00258167	0.003603134
**K03.5**	**Ankylosis of teeth**	0.0060428	0.00526343	0.00469197	0.004745214	0.00322709	0.004804179
**K03.2**	**Erosion of teeth**	0.0054067	0.00433459	0.00500477	0.003796171	0.00258167	0.004235263
**K10.8**	**Other specified diseases of jaws**	0.0054067	0.0068115	0.00469197	0.005061561	0.01032668	0.006447714
**K06.9**	**Disorder of gingiva and edentulous alveolar ridge, unspecified**	0.0050887	0.00650189	0.00594316	0.006326952	0.00516334	0.005815585
**K03.8**	**Other specified diseases of hard tissues of teeth**	0.0047706	0.0068115	0.00594316	0.005694256	0.0074223	0.00613165
**Z97.2**	**Presence of dental prosthetic device (complete)(partial)**	0.0047706	0.00495382	0.00656876	0.006959647	0.01064939	0.006763779
**K13.1**	**Cheek and lip biting**	0.0047706	0.00619228	0.00437917	0.006326952	0.00484063	0.005309882
**K03.3**	**Pathological resorption of teeth**	0.0044526	0.0021673	0.00563037	0.006326952	0.00548605	0.004804179
**K11.6**	**Mucocele of salivary gland**	0.0041345	0.00123846	0.00344078	0.002847128	0.00129083	0.002591728
**G50.0**	**Trigeminal neuralgia**	0.0038165	0.00061923	0.00093839	0.000949043	0.00032271	0.001327471
**K13.7**	**Other and unspecified lesions of oral mucosa**	0.0038165	0.00588266	0.00969674	0.00917408	0.00935855	0.007585546
**Z46.3**	**Fitting and adjustment of dental prosthetic device**	0.0034984	0.00433459	0.00437917	0.004112519	0.0038725	0.004045625
**K13.6**	**Irritative hyperplasia of oral mucosa**	0.0028624	0.00371537	0.00406638	0.001898085	0.0038725	0.00328707
**Z96.5**	**Presence of tooth-root and mandibular implants**	0.0028624	0.00278652	0.01313752	0.019297202	0.0148446	0.010556552
**K07.2**	**Anomalies of dental arch relationship**	0.0028624	0.00309614	0.00218959	0.002530781	0.00484063	0.003097431
**S00.5**	**Superficial injury of lip and oral cavity**	0.0028624	0.00247691	0.00312798	0.002530781	0.00225896	0.002654941
**K13.4**	**Granuloma and granuloma-like lesions of oral mucosa**	0.0025443	0.00371537	0.00187679	0.002530781	0.00451792	0.003034218
**K13.2**	**Leukoplakia and other disturbances of oral epithelium, including tongue**	0.0022263	0.00495382	0.00312798	0.001581738	0.00258167	0.002907793
**K03.7**	**Posteruptive colour changes of dental hard tissues**	0.0022263	0.00123846	0.00250239	0.000632695	0.00096813	0.001517109
**K09.8**	**Other cysts of oral region, not elsewhere classified**	0.0022263	0.00154807	0.00344078	0.002847128	0.00258167	0.002528515
**K00.4**	**Disturbances in tooth formation**	0.0019082	0.00185768	0.00344078	0.003479823	0.00225896	0.002591728
**K09.1**	**Developmental (nonodontogenic) cysts of oral region**	0.0015902	0.00309614	0.00406638	0.00126539	0.00322709	0.002654941
**K03.4**	**Hypercementosis**	0.0015902	0.00154807	0.00156399	0.000949043	0.00225896	0.001580322
**K07.0**	**Major anomalies of jaw size**	0.0015902	0.00154807	0.00125119	0.000316348	0.00322709	0.001580322
**B00.2**	**Herpesviral gingivostomatitis and pharyngotonsillitis**	0.0012722	0.00092884	0.0006256	0.00126539	0.00161354	0.001137832
**K02.4**	**Odontoclasia**	0.0012722	0.00154807	0.0006256	0.000316348	0.00064542	0.00088498
**K07.8**	**Other dentofacial anomalies**	0.0009541	0.00154807	0.0006256	0.00126539	0.00032271	0.000948193
**K00.2**	**Abnormalities of size and form of teeth**	0.0006361	0.00061923	0.0006256	0.000316348	0.00096813	0.000632129
**T49.7**	**Dental drugs, topically applied**	0.0006361	0.00061923	0.00093839	0.000316348	0.00096813	0.000695342
**K07.1**	**Anomalies of jaw–cranial base relationship**	0.0006361	0.0021673	0.00187679	0.004112519	0.00129083	0.002022812
**L43**	**Lichen planus**	0.0006361	0.00061923	0.00125119	0.000632695	0.00225896	0.001074619
**K07.9**	**Dentofacial anomaly, unspecified**	0.0006361	0.00185768	0.00406638	0.003163476	0.00258167	0.002465302
**K00.5**	**Hereditary disturbances in tooth structure, not elsewhere classified**	0.000318	0.00030961	0.0006256	0.000949043	0.00032271	0.000505703

**Table 9:** List of indications in which antibiotics may or may not be prescribed.

		Percentage Out of Total Drug Prescription					
ICD 10 Code	Indication	2015	2016	2017	2018	2019	Total
**K04**	**Diseases of pulp and periapical tissues**	15.636479	14.6506163	14.6195593	14.87308135	14.6212852	14.88947859
**K04.4**	**Acute apical periodontitis of pulpal origin**	6.5570486	6.69292192	6.95882013	6.817922988	6.47644065	6.707013343
**K12**	**Stomatitis and related lesions**	0.1784209	0.13158587	0.15514788	0.20246245	0.19039813	0.171433339
**S03.2**	**Dislocation of tooth**	0.0108134	0.00804996	0.00938394	0.010755818	0.00806772	0.00941872
**K14**	**Diseases of tongue**	0.0031804	0.00433459	0.00250239	0.005061561	0.00322709	0.003666347
**K11.8**	**Other diseases of salivary glands**	0.0019082	0.00123846	0.00093839	0.002847128	0.00225896	0.001833174
**S01.5**	**Open wound of lip and oral cavity**	0.0019082	0.00185768	0.00187679	0.003163476	0.00161354	0.002086025

## Data Availability

The data presented in this study are available on request from the corresponding author. The data are not publicly available unless permission is obtained from regulatory institutions.
